# Defining rights-based indicators for HIV epidemic transition

**DOI:** 10.1371/journal.pmed.1002720

**Published:** 2018-12-21

**Authors:** Joseph J. Amon, Patrick Eba, Laurel Sprague, Olive Edwards, Chris Beyrer

**Affiliations:** 1 Office of Global Health and Department of Community Health and Prevention, Dornsife School of Public Health, Drexel University, Philadelphia, Pennsylvania, United States of America; 2 UNAIDS, Bangui, Central African Republic; 3 Global Network of People Living with HIV, Amsterdam, the Netherlands; 4 Jamaica Community of Positive Women/ICW Caribbean, St. Catherine, Jamaica; 5 Center for Public Health and Human Rights, Department of Epidemiology, Johns Hopkins Bloomberg School of Public Health, Baltimore, Maryland, United States of America

## Abstract

In a Policy Forum, Joseph Amon and colleagues discuss human rights indicators for tracking progress toward ending the HIV epidemic.

**Summary points**

- Stark differences in HIV incidence and mortality across locations and populations demonstrate the challenge of identifying a single indicator, at national scale, of progress toward the control of HIV epidemics.
- Even in countries that report decreases in HIV incidence, incidence may be increasing among groups that are particularly vulnerable and face political and social exclusion, especially sex workers, people who inject drugs, transgender persons, men who have sex with men, and prisoners.
- To comprehensively evaluate national progress on HIV, five categories of indicators should be examined that address: levels of coverage of key evidence-based prevention and treatment interventions; incidence and prevalence of HIV infection; AIDS-related or all-cause mortality among people living with HIV; stigma and discrimination; and the legal and policy environment.
- Indicators should be disaggregated, whenever feasible, to fully reflect progress and challenges relating to all populations and locations in the national response.
- The process of evaluating indicators of national progress should meaningfully involve people living with HIV and from key populations as an important part of data validation.

In 1971, Omran described a sweeping theory of epidemic transition: as countries become wealthier, modernization and socioeconomic progress drive down high rates of mortality due to infectious diseases while noncommunicable disease (NCD) burdens increase [1]. Although the theory was initially met with support, the spread of HIV in high-income countries and the growing burden of NCDs in low- and middle-income countries, along with the recognition of health inequities in both settings, raised doubts about the validity of a single, generalizable theory of epidemic transition.

In the past few years, UNAIDS, the United States President’s Emergency Plan for AIDS Relief (PEPFAR), and The Global Fund to Fight AIDS, Tuberculosis and Malaria (The Global Fund) have promoted a goal of HIV epidemic transition and an “end of AIDS.” The belief in this goal stems from the significant advances, nearly four decades since the first report of AIDS, in treatment for HIV infection, community mobilization, and political will. Reflecting this optimism, in October 2017 UNAIDS sponsored a meeting of global experts to find a consensus around a single indicator that could define, at national scale, “epidemic control,” generally understood as a shift from high HIV incidence and mortality to low levels of transmission and effectively managed care [2–3].

However, as with Omran’s theory, a closer look at the diversity of local experiences reveals a more complex reality. Progress on HIV and AIDS to date is markedly uneven, with 1.8 million new HIV infections in 2017 and stark disparities in incidence and mortality among different populations and locations within countries. Whereas the importance of measuring our success in controlling the HIV epidemic is uncontestable, discussion around the end point and possible indicators at the UNAIDS meeting was hotly contested. Our approach in this article, reflecting the social, political, and structural factors that shape risk and influence access to prevention and care, calls for the use of a collection of indicators that incorporate principles of human rights, gender equality, and participation, recognizing that without equity, epidemic transition cannot truly be achieved or sustained.

## Political epidemiology of HIV

From early in the global HIV epidemic, we have understood that national indicators of HIV incidence and prevalence represent a sum of the diverse subepidemics affecting different populations according to geography, socioeconomic status, gender, criminalized status, acquisition risks, and vulnerability to rights abuses. Even in countries that report decreases in HIV incidence, incidence may be increasing among groups that are particularly vulnerable and face political and social exclusion, especially sex workers, people who inject drugs (PWID), transgender persons, gay men and other men who have sex with men (MSM), and prisoners (known collectively as key populations) [4].

Knowledge of HIV testing and access to treatment among key populations remain low in many regions, and discrimination, criminalization, violence, and other human rights violations are common. Punitive laws, policies, and practices targeting key populations and people living with HIV—and outreach workers working with these populations—increase vulnerability to HIV infection, limit access to care, and threaten lives [5]. For example, in Chechnya in 2017, a brutal campaign by law enforcement and security agency officials targeted LGBT individuals, rounding up dozens of men on suspicion of being gay, and torturing and humiliating the victims [6]. In the Philippines, more than 12,000 individuals suspected of using drugs have been murdered in state-sponsored extrajudicial killings [7]. In Belarus, in the first 6 months of 2017, more than 50 people living with HIV were prosecuted for criminal HIV transmission after a spouse or partner registered for clinical HIV care, despite the lack of evidence regarding the direction of transmission, disclosure, or consent [8].

Even in countries reporting progress in addressing HIV, key populations are often left behind. In 2016, Thailand was certified free of perinatal transmission of HIV infection [9]. Rates of infection in women of childbearing age, in pregnant women, and in the perinatal period have been in steady decline for more than a decade [10]. Yet, over this same period, HIV incidence has risen steadily among MSM and transgender women who have sex with men. In the subset of MSM who sell sex in Thailand, HIV burdens are even higher, and the country has done little to promote access to pre-exposure prophylaxis (PrEP) among MSM, despite demonstrated efficacy since 2011 [10–11].

The severe and expanding epidemic of HIV-1 across the Russian Federation is another example of a policy and prevention failure. Three pillars define HIV prevention for PWID: harm reduction with needle and syringe access, evidence-based treatment for drug dependency, and antiretroviral (ARV) therapy for those living with HIV infection. Yet none of these pillars are widely available in the Russian Federation [12]. Medication-assisted therapy with methadone or buprenorphine remains illegal in Russia. Harm reduction had been supported by The Global Fund, but, in the transition to Russian national resources, harm reduction programs have drastically declined. And limitations on ARV access for those with current or past histories of substance use have meant that Russian PWID have extraordinarily low rates of ARV treatment access. Taken together, these failures of policy and practice have led to the largest HIV epidemic in Europe and one of the only expanding epidemics in a developed country.

Until recently, Tanzania’s response to HIV was considered one of the most effective and inclusive in sub-Saharan Africa. Between 2000 and 2016, new HIV infections in the country decreased by more than half. Coverage of ARV therapy increased from 18% to 62%, and AIDS-related deaths declined from 120,000 in 2005 to fewer than 34,000 in 2016 [13]. In its national HIV plan, Tanzania recognized the vulnerability to HIV among key populations as well as the specific legal and social challenges that they face [14]. However, recent changes in leadership in the country are eroding the advances made in the response to the epidemic. References to key populations and to evidence-informed services to address their needs have been removed from HIV strategy documents. In the past 2 years, HIV prevention services such as lubricants for gay men and MSM, as well as needle exchange for PWID, have been terminated or prohibited. Nongovernmental organizations (NGOs) and community leaders working with these populations are harassed, arrested, prosecuted, or forced to flee [15].

In addition to attention to key populations, effective HIV responses require attention to locations. Data from Kenya show that 65% of all new HIV infections in the country occurred in only 9 of 47 counties [16]. Canada’s Saskatchewan province has triple the HIV incidence of the rest of Canada, and 79% of people newly diagnosed are indigenous peoples [17]. In the West and Central African regions, a complex panorama of weak health systems, low prioritization of HIV, prevalent stigma, limited civil society capacity, and prevailing humanitarian crises have contributed to slower progress against the HIV epidemic, with high HIV-related mortality and lower coverage of ARV treatment [18]. These realities of populations and locations left behind, as well as the political and legal determinants structuring risk and vulnerability, represent the political epidemiology of HIV epidemics [19] and require attention in any measure of transition to an end of the global AIDS epidemic.

## Measuring epidemic transition

In the past 100 years, three disease eradication programs have failed (malaria, yellow fever, and yaws), two are ongoing (guinea worm and polio), and one has been successful (smallpox) [20]. A common cause for failure was inadequate attention to social and political context. Whereas epidemic transition may present a lower bar then eradication or disease elimination, the need to address sociopolitical context remains, and the definitions of indicators in the elimination of vertical HIV transmission and of certain neglected tropical diseases (NTDs) provide useful examples of more-comprehensive approaches.

In 2014, WHO, together with the Global Network of People Living with HIV and the International Community of Women Living with HIV, initiated a process for validation of EMTCT [21]. In addition to traditional epidemiological indicators, countries were required to document that policies had been adopted to eliminate the criminalization of vertical transmission; mandatory or coerced testing and treatment; forced and coerced abortion, contraception, and/or sterilization; and that access to justice was possible for those facing rights violations [22]. The validation process emphasized community engagement and specified that a country that meets all biomedical requirements may nonetheless not be validated if there is evidence of “grave or systematic recent or ongoing human rights violations” [23].

The criteria for the elimination of NTDs as a public health problem, set forth by WHO in 2015, are less expansive in addressing human rights considerations [24]. However, criteria for trachoma and lymphatic filariasis elimination, for example, include specific considerations related to comprehensive prevention interventions, access to care, and health system strengthening.

In this context, five categories of indicators should be included in any overall consideration of the validation of successful, sustained, and equitable HIV epidemic transition (see Table 1).

**Table 1:** Rights-based evidence and indicator framework for epidemic transition.

Category	Epidemic Transition Indicators
Implementation	• Coverage level of key evidence-based prevention and treatment interventions per population and location and over time
Disease	• Incidence/prevalence of HIV infection for key populations • AIDS-related or all-cause deaths among PLHIV
Access to Treatment	• Disaggregated treatment coverage and viral load for key populations • Treatment coverage in children and adolescents • Person-days, by district, of lack of availability of ARVs due to stock-outs or other interruption in supply
Stigma and Discrimination	• Experience of HIV-related discrimination in healthcare settings • Workplace HIV discrimination • Avoidance of healthcare among key populations because of stigma and discrimination
HSS/Legal and Policy Framework	• Existence of discriminatory laws and policies: ○ criminalization of key populations ○ overly broad HIV exposure and transmission laws ○ compulsory detention without due process for people who use drugs and sex workers ○ mandatory or coerced HIV testing and treatment ○ forced sterilization • Adoption of nondiscriminatory policies in health settings, promotion of “patients’ rights,” and mechanisms for redress • Country investment in HIV response in all affected communities • Community engagement and participation

Abbreviations: ARV, antiretroviral; HSS, health system strengthening; PLHIV, people living with HIV.

First, efforts to assess epidemic transition should include documentation of the implementation of comprehensive, evidence-informed, and rights-based HIV prevention, treatment, and care programs for all populations. This should include, for example, data on the availability and uptake of harm reduction programs for people who use drugs, mental health and social and community support programs, and condoms and other prevention and treatment programs in prisons and detention centers, as well as structural interventions such as educational opportunities for adolescent girls and young women and social protection policies for vulnerable groups.

Second, transition indicators relating to incidence and prevalence as well as mortality should be disaggregated, whenever feasible, to fully reflect progress and challenges relating to all populations and locations in the national response.

Third, data on treatment coverage, stock-outs, or other structural factors of treatment interruption, as well as viral suppression, should be measured and similarly disaggregated. Many countries currently do not collect or report accurate (or any) data on the impact of the epidemic on key populations and their access to HIV services, despite the reality that between 40% and 50% of all new HIV infections among adults worldwide occur among people from key populations and their immediate partners [25].

Fourth, epidemic transition data must involve the measurement of stigma and discrimination. Related, in a fifth category, is the identification and monitoring of laws, policies, and practices that violate human rights and that make people vulnerable to HIV or hinder their access to prevention, treatment, and care. These may include HIV criminalization; coerced HIV testing or treatment; forced abortion, contraception, or sterilization; discrimination in healthcare settings; and the criminalization of key populations [26].

Nearly all of the indicators proposed to assess stigma and discrimination in HIV responses are currently collected using routine surveillance or special studies (for example, the Global AIDS Monitoring [GAM] tool [27], the People Living With HIV Stigma Index [28], biobehavioral surveys [29], and the National Commitments and Policy Instrument [27]).

## Community participation in defining and measuring epidemic transition

A hallmark of the HIV response, and a major factor in its success to date, has been the meaningful involvement of affected communities generally, and people living with HIV specifically (often referred to as the greater involvement of people living with HIV or AIDS [GIPA]) [30]. This participation—at all levels, from the local design and implementation of programs to the global governance of such institutions as UNAIDS and The Global Fund—has contributed to effective responses and attention to gaps and challenges that would otherwise be overlooked.

The engagement of civil society organizations and affected populations has also been an essential part of monitoring the epidemic and holding governments accountable that have denied the existence of HIV, persecuted key populations, or adopted ideological rather than evidence-informed approaches [31]. One means of this monitoring has been the involvement of civil society and community actors in the collection, interpretation, and reporting of national data and indicators related to the 2001, 2011, and 2016 UN Declarations on HIV/AIDS [32]. Recently, civil society and people living with HIV in countries such as South Africa and Cameroon have established community observatories that provide real-time and reliable data on HIV treatment stock-outs, quality of care, and discrimination and ill-treatment in health facilities [33].

The process of defining a meaningful measure of HIV epidemic transition must consider both who defines epidemic transition and who assesses epidemic transition. Any validation process for epidemic transition should meaningfully involve and fund people living with HIV and people from key populations to conduct community monitoring and participate in committees responsible for assessing HIV epidemic transition at all levels.

## Conclusion

The world is ready to celebrate an “end of AIDS.” It is important to recall, however, that past efforts to define epidemic control may have done more harm than good, undermining realistic planning and policy making. To combat this, a broader, rights-based indicator framework should be at the center of what epidemic transition means. This rights-based indicator framework should both track the factors driving the HIV epidemic and measure our progress from a highly stigmatized epidemic that generates discrimination and human rights abuses to a rational, evidence-informed, and rights-based response that respects the dignity and rights of those living with and vulnerable to HIV.

## Abbreviations

ARV: antiretroviral
GAM: Global AIDS Monitoring
GIPA: the greater involvement of people living with HIV or AIDS
MSM: men who have sex with men
NCD: noncommunicable disease
NGO: nongovernmental organization
NTD: neglected tropical diseases
PEPFAR: United States President’s Emergency Plan for AIDS Relief
PLHIV: people living with HIV
PrEP: preexposure prophylaxis
PWID: people who inject drugs
